# Bioprinting of Collagen Type I and II *via* Aerosol Jet Printing for the Replication of Dense Collagenous Tissues

**DOI:** 10.3389/fbioe.2021.786945

**Published:** 2021-11-05

**Authors:** Rory Gibney, Eleonora Ferraris

**Affiliations:** ^1^ Department of Mechanical Engineering, KU Leuven Campus De Nayer, Leuven, Belgium; ^2^ Department of Materials Engineering, KU Leuven, Leuven, Belgium

**Keywords:** bioprinting, 3D printing, biomaterials, connective tissue, collagen type II, collagen type I, viscosity, nanoindentation

## Abstract

Collagen has grown increasingly present in bioprinting, however collagen bioprinting has mostly been limited to the extrusion printing of collagen type I to form weak collagen hydrogels. While these weak collagen hydrogels have their applications, synthetic polymers are often required to reinforce gel-laden constructs that aim to replicate dense collagenous tissues found *in vivo*. In this study, aerosol jet printing (AJP) was used to print and process collagen type I and II into dense constructs with a greater capacity to replicate the dense collagenous ECM found in connective tissues. Collagen type I and II was isolated from animal tissues to form solutions for printing. Collagen type I and II constructs were printed with 576 layers and measured to have average effective elastic moduli of 241.3 ± 94.3 and 196.6 ± 86.0 kPa (±SD), respectively, without any chemical modification. Collagen type II solutions were measured to be less viscous than type I and both collagen type I and II exhibited a drop in viscosity due to AJP. Circular dichroism and SDS-PAGE showed collagen type I to be more vulnerable to structural changes due to the stresses of the aerosol formation step of aerosol jet printing while the collagen type II triple helix was largely unaffected. SEM illustrated that distinct layers remained in the aerosol jet print constructs. The results show that aerosol jet printing should be considered an effective way to process collagen type I and II into stiff dense constructs with suitable mechanical properties for the replication of dense collagenous connective tissues.

## Introduction

Collagens are a family of extracellular matrix proteins found in all metazoan ECM from sponges to humans. The first member of the collagen family (a collagen type IV variant) is thought to have evolved over 700 million years ago in the last common ancestor between metazoa, choanoflagellates and filasterean, and is regarded by some as having facilitated multicellularity (Fidler et al., 2018). From this first member of the collagen family the other members are thought to have evolved, which currently rests at 28 different collagens in mammals (Karsdal, 2016). All collagens possess a characteristic right-handed triple helix structure which is assembled by three polypeptide chains, called α-chains, with left-handed polyproline II helices (Adzhubei et al., 2013). The α-chains may be identical, like homotrimeric collagen type II, or a mix of two or more genetically distinct α chains, like heterotrimeric collagen type I. Collagens type I and type II are members of a collagen sub-family called the fibrillar collagens which are the most common collagens in mammals and are responsible for most of the stereotypical collagen properties, such as, the ability to self-assemble into fibrils with D-banding. They are also used to make collagen hydrogels which are a very attractive tool for tissue engineering given their ability to mimic native ECM for a wide range of tissues (Sander and Barocas, 2008). The formation of collagen hydrogels is based on the self-assembly of fibrils *in vitro*, which is mainly controlled by pH, ionic strength, and temperature (Gobeaux et al., 2008; Li et al., 2009). Native type fibrils with characteristic D-banding begin to form *in vitro* within the pH range of 5.0–8.5, ionic strength between 0.1 and 0.8, and temperatures from 4 to 37°C (Li et al., 2009; Xie et al., 2017). There is a close relationship between time and temperature in respect to fibril formation, with lower incubation temperatures requiring much longer incubation times; around 15 h at 4°C compared to around 30 min or more at 37°C (Xie et al., 2017). When casting collagen gels this incubation period does not pose much of a problem since multiple samples can be cast simultaneously, meaning the main limitation on production is the number of available casts. However, when considering the layer-by-layer building process of additive manufacturing, this incubation step makes collagen less attractive if it must be implemented between layers.

Bioprinting has been facilitated by a number of additive manufacturing technologies. These are typically either extrusion-based, droplet based, or light based. Extrusion based methods usually extrude a hydrogel or hydrogel precursor through a nozzle *via* pneumatic or mechanical means (Ozbolat and Hospodiuk, 2016). Droplet, or jet-based methods, similar to conventional inkjet printing, generally use less viscous inks and eject droplets of the ink from a reservoir through a nozzle onto a substrate, facilitated by a stimulus in the printhead such as heat or piezoelectric actuation (Saunders and Derby, 2014; Gudapati and Ozbolat, 2020). The most common light based additive manufacturing methods used in bioprinting are vat polymerization methods which contain the ink in a transparent vat and use light to polymerize monomers within the ink *via* a photoinitiator (Ng et al., 2020). All these technologies execute code which contains the geometry of a virtual 3-dimensional object in 2-dimensional slices, or layers, which are executed consecutively to build the physical 3D object. Naturally, this relies on some form of phase transition from a fluid to some form of a solid, often a hydrogel. This phase transition can be an inherent feature of the material, like the self-assembly of collagen molecules into fibrils, or it can be the result of chemical modification of the material, such as the photocrosslinking of collagen methacrylamide (Drzewiecki et al., 2017). Most examples of collagen printing are extrusion methods that rely on collagen fibrillogenesis. However, the slow solution-to-gel transition of collagen fibrillogenesis allows time for a deposited print line to flow and spread away from its deposited position, which results in poor print resolution, and can make multiple layers difficult to achieve (Murphy et al., 2013). Innovative methods have managed to circumvent these limitations such as microvalve printing multiple thin layers of collagen with a nebulized alkaline solution providing a coating between layers to raise the pH and allow fibrillogenesis (Lee et al., 2009). For extrusion printing, it was found that at a high enough concentration and suitable pH the viscosity of a collagen solution can make it feasible to print multiple over-lying layers at room temperature with an incubation step at 37°C post-print rather than between layers (Diamantides et al., 2017). This understanding of the ideal rheology of a collagen solution for printing has been complemented by new extrusion printing techniques. Printing methods, such as the FRESH method, have further improved the accuracy and resolution of collagen printing (Park et al., 2014; Yoon et al., 2016; Lee et al., 2019a, 2021). However, the mechanical properties of printed collagen constructs remains limited when compared to the dense collagenous tissues from which the collagen is typically sourced and the replication of which is a often attempted. This can be attributed to the low range of collagen concentrations that are suitable for most printing techniques, generally <2% w/v (Diamantides et al., 2017). Whereas, collagen concentration in native dense collagenous tissues is often much higher ranging from >10% in cartilage, to >14% in the cornea (Leonard and Meek, 1997; Fox et al., 2009). At higher concentrations collagen becomes too viscous for extrusion or droplet methods, and likely too turbid for light based methods. Some chemical crosslinking techniques can greatly improve the mechanical properties of a printed scaffold, but this can also result in cytotoxicity, and/or the consumption of motifs that are involved in integrin-mediated cell binding (van Luyn et al., 1992; Bax et al., 2019).

Collagen printing thus far has focused almost exclusively on collagen type I, likely due to its commercial availability, particularly with the arrival of commecial collagen type I inks for extrusion bioprinting. However other fibrillar collagens such as collagen type II could prove useful particularly in cartilage tissue engineering. Collagen type II doesn’t have the same commercial availability as collagen type I, and collagen type II is also associated with the induction of arthritis in rats, however this association is related to the intravenous administration of soluble (monomeric) collagen type II, and can be treated prophylactically (Morgan et al., 1980; Nagler-Anderson et al., 1986; Mikulowska et al., 1997). Collagen type II has been shown to improve attachment of mesenchymal stem cells (MSCs), and induce and maintain MSC chondrogenesis when used in TE scaffolds (Bosnakovski et al., 2006; Ragetly et al., 2010). Collagen type II has also been shown to perform similar to type I in comparative chondrogenic studies (Freyria et al., 2009; Rutgers et al., 2013). While commercial collagen type II might be less common, the extraction of collagen type II is very similar to that of collagen type I with the only major difference being the starting tissue, which is typically articular cartilage in place of the tendon, bone or skin that is typically used for the extraction of collagen type I. Therefore by investigating the bioprinting of collagen type II some of the advantages of collagen type I as a substrate for cell culture could be exploited with a different set of material properties for more bioprinting options.

**FIGURE 1:**
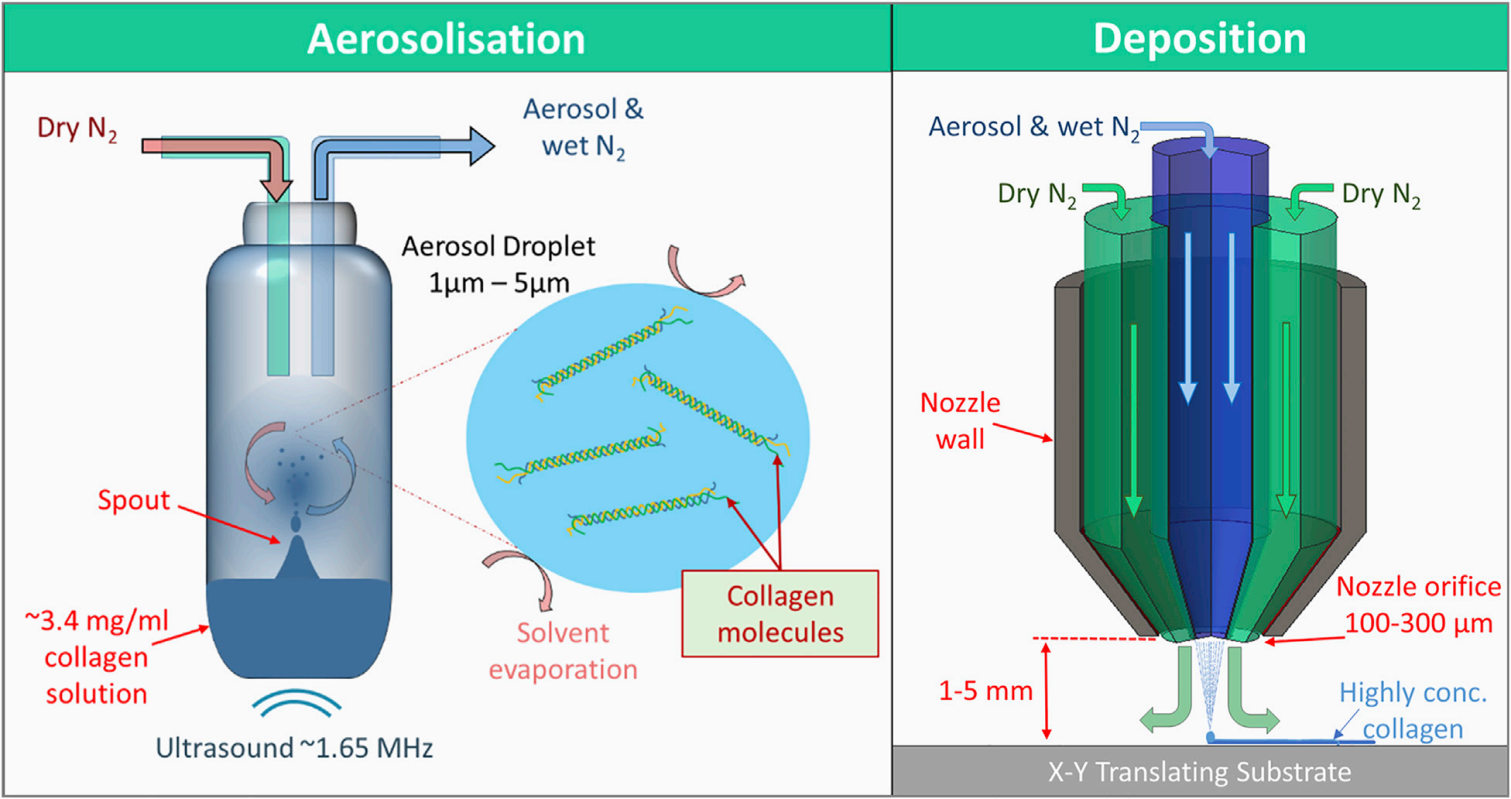
The two major steps of aerosol jet printing. Firstly, aerosolization, where 1.65 MHz ultrasound generates a standing wave within the ink reservoir resulting in the formation of a spout, the surface of which resonates and disperses aerosol-sized (<5 µm diameter) droplets of ink. The droplets then mix and become entrained with a flow of nitrogen gas through the reservoir towards the deposition head. Secondly, in the deposition head, the aerosol-laden nitrogen is collimated by a secondary annular flow of nitrogen, separating the aerosol flow from the walls of the nozzle. The aerosol droplets then coalesce on an x-y translating print substrate. The collimation of the aerosol continues for up 5 mm after the nozzle. The high surface area to volume ratio of the aerosol and flow of dry nitrogen gas allows the ink to dry very quickly in transit and on the substrate resulting in highly concentrated printed collagen.

Aerosol jet® printing (AJP) is a printing method that forms an aerosol from an ink and carrier gas, and forces the aerosol to coalesce on a substrate *via* impaction. It was developed as a way to print electronic components on to virtually any surface topography, which has made some impact in bio-electrical applications (Sarreal and Bhatti, 2020; Seiti et al., 2020). AJP has also been investigated for the deposition of DNA, enzymes, and silk fibroin with moderate success (De Silva et al., 2006; Grunwald et al., 2010; Williams et al., 2019; Xiao et al., 2020). AJP could be considered a droplet or jet-based method similar to inkjet, yet it is very different. By forming an aerosol from the ink, AJP exploits some of the unique properties of aerosol droplets during their brief transit from the ink reservoir to the substrate. Aerosol (liquid) droplets have a very high surface area to volume ratios which can facilitate a high rate of solvent evaporation/diffusion from the droplet, as seen in Figure 1 (Bzdek et al., 2020). Aerosol droplets can also reach solute concentrations that are much higher than the saturation concentration of bulk solutions (Yan et al., 2016; Bzdek et al., 2020). Hence a dilute collagen solution can be deposited as highly concentrated collagen *via* AJP. This is advantageous since viscosity is a limiting factor for AJP inks with inks typically in the range of 1–10 mPa s in viscosity (for ultrasonic atomization). As the ink aerosol approaches the nozzle it is confined and collimated into a dense stream of aerosol droplets by a coaxial annular flow of gas (sheath gas). The velocity and subsequent inertia of the droplets in this collimated stream, or aerosol jet, allows for the nozzle of the printer to be up to 5 mm away from the substrate, which facilitates the printing onto many different surfaces. As a result of the sheath gas the nozzle diameter does not equate to the print resolution. Printed lines or features can be printed that are 90% smaller [as low as 10 µm (Cai et al., 2016)] than the nozzle orifice diameter, which ranges from 100 to 300 µm in multiples of 50 µm. This helps prevent nozzle clogging and removes some of the complications regarding fabrication, handling and cleaning of a much smaller nozzle.

For the aerosol jet printer used in this work, the Optomec AJ300, the ink reservoir must be constantly radiated with ultrasound from a 1.65 MHz piezoelectric diaphragm during printing. The thermal stresses that arise from the ultrasound are somewhat mitigated by cooling of the atomizer, however there is no mitigating factor for the physical stress of the ultrasound and atomization. Sound and ultrasound has been shown to cause degradation of collagen type I into shorter triple helical fragments with the length of the triple helical fragments decreasing with increasing exposure (Nishihara and Doty, 1958; Giraud-Guille et al., 1994). The effects of sonic-induced heating must be mitigated to preserve the triple helix, however the thermal stability of the triple helix also decreases as it becomes more fragmented (Giraud-Guille et al., 1994). Nevertheless, this sonic fragmentation of collagen type I has been exploited to enhance the formation of dense tissue-like liquid crystalline collagen assemblies (Giraud-Guille, 1989; Mosser et al., 2006). This enhancement is suggested to be the result of the ultrasonic fragmentation causing a drop in viscosity of the collagen solution, and allowing a distribution of shorter collagen molecules to provide less obstruction to the alignment of the collagen molecules forming liquid crystalline assemblies quicker than non-sonically fragmented solutions (Giraud-Guille, 1989). Therefore, despite the potential of this printing technique to degrade collagen, it could provide a way to print liquid crystalline assemblies of collagen, or at least print very dense collagen constructs with the potential to replicate highly collagenous tissues.

## Materials and Methods

### Collagen Extraction

Collagen extractions were performed using protocols with some modifications (Zeugolis et al., 2008; Delgado et al., 2017; Sorushanova et al., 2021). Briefly, frozen bovine Achilles tendons were defrosted, fascia were trimmed, and the tendons cut into 1 cm cubes. The tendons were frozen and cryo-milled. The milled tendons were washed in 1XPBS at 4°C three times, after each wash the suspension was centrifuged and the supernatant discarded. The milled tendon was then re-suspended in 0.5 M acetic acid at 4°C using an overhead mixer over 48 h. Pepsin was then added to the suspension at a rate of 1 g per 100 g of wet tendon and left for another 72 h. The suspension was then filtered through a 100 µm sieve. Salt was then added to reach a 0.9 M NaCl solution and the collagen precipitated. Collagen fibrils were collected with a sieve and centrifuged to remove any remaining solution. The collagen fibrils were re-suspended in 1 M acetic acid at 4°C, then centrifuged, and any precipitated material was discarded. The purified collagen solution was put into dialysis tubing and dialyzed against 1 mM acetic acid, with the dialysate changed 5 times. The purity of the dialysed collagen solution was assessed by SDS-PAGE with silver staining, then frozen and lyophilized.

This process was repeated using porcine articular cartilage for the extraction of collagen type II. In brief, slices of cartilage were peeled from the femoral condyles and femoral head of porcine femurs using a scalpel. Resected cartilage pieces were immediately placed in 1XPBS on ice. The cartilage was then frozen and cryo-milled. All steps thereafter were identical to the collagen type I extraction. Collagen inks were made by adding the lyophilised collagen type I or type II to a suitable amount of cold 0.01 M hydrochloric acid and stirring at 4°C for at least 48 h.

### Aerosol Jet Printing of Collagen Type I and II

All printing was performed using an Optomec^®^ AJ300 aerosol jet printer. Six sets of samples were printed from 3 different inks, 3 mg/ml collagen type I, and 3 mg/ml & 6 mg/ml collagen type II, and 2 different print programs (A and B). Collagen type II solutions were clearly less viscous that type I solutions, hence the use of 6 mg/ml collagen type II while no higher concentrations of collagen type I could be printed in preliminary experiments. Both print program A and B consisted 576 layers of rectilinear rasters (or serpentine infill) of a circle, where each even-numbered layer was rotated 90° relative to the pattern of the previous layer, and each odd-numbered layer was rotated by 15° relative to the last odd-numbered layer. This regime was previously developed to avoid overlap of peaks and troughs and produce relatively flat samples. The programs were inherently different and were executed with different parameters, summarized in Table 1. Program A was for a 4 mm sample and the spacing between parallel print lines was 60 µm. Program A was executed with a 100 µm nozzle, 50 ml/min atomizer flow, 50 ml/min sheath flow, and a print speed of around 12.5 mm/s which typically resulted in print lines of 60–65 µm in width leading to continuous films as layers. Program B was for a 5 mm sample and the spacing between parallel print lines was 90 µm. Program B was executed with a 150 µm nozzle, 50 ml/min atomizer flow, 100 ml/min sheath flow, and a print speed of around 17.5 mm/s, which typically resulted in print lines of 60–70 µm, leading to more of a lattice structure. All samples were printed onto glass cover slips that had been washed for 12 h in 20% v/v nitric acid. In all cases print parameters were varied slightly during printing to account for changes in aerosol production, and the volume of ink in the vial was replenished with every hour of printing. This particular regime was selected as the print time and aerosol flow were similar for both samples despite the discrepancy in size. It was also desired to increase the sample surface area improve the likely outcome of cell-seeding and maximise the possible cell number. Hence, program B was developed to compare to program A, which had typically been typically employed for printing collagen. Once printed, the samples were stored in a well plate at 4°C to await processing.

**TABLE 1:** Print parameters of samples printed with program A and program B.

Parameter	Program A	Program B
Sample diameter	4.5 mm	5.25 mm
Track spacing	60 µm	90 µm
Atomizer flow	50 ml/min	50 ml/min
Sheath flow	50 ml/min	100 ml/min
Print velocity	12.5 mm/s	17.5 mm/s
Exp. Print line width	60–65 µm	60–70 µm
Exp. print pressure	0.4–0.6 bar	0.1–0.2 bar

#### Post Print Processing of Aerosol Jet Printed Collagen Type I and II

**FIGURE 2:**
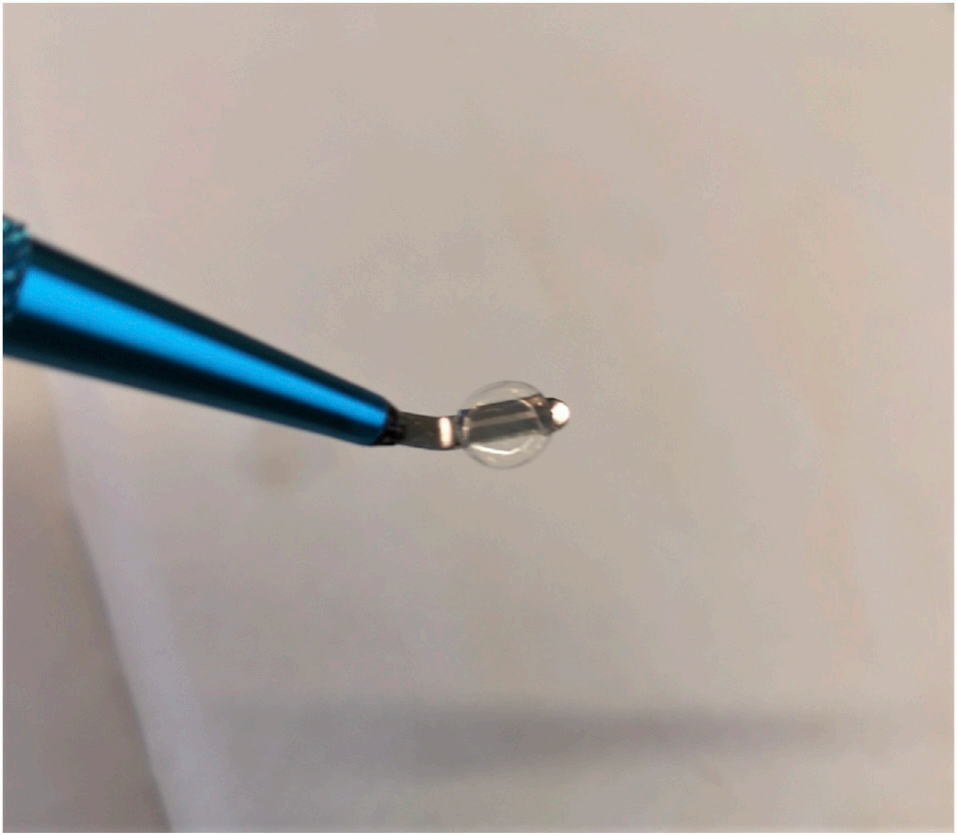
A 6 mg/ml collagen type II 5.25 mm diameter sample after being removed from its print substrate post neutralization.

Since all samples were printed from an acidic collagen solution, the samples possessed an inherent acidity and upon incubation in aqueous media residual solvent in the printed material would leach out leading to a low pH and re-solubilization of the material. Hence a neutralizing buffer was used which employed ethanol to slow the hydration of the printed material to allow time for any leachate to be neutralized, thereby preventing re-solubilization of the printed material. The neutralizing buffer consisted of 3 parts ethanol and 2 parts 1XPBS. Prior to mixing of the two parts of the neutralizing buffer, the pH of the PBS was adjusted to pH 8-9 with 1 M NaOH. Thereafter the ethanol was slowly added to the PBS while stirring, and left stirring for approximately 1 h. The pH was checked again at this point and adjusted as necessary to pH 8-9 with 1 M NaOH. The buffer was then stored at 4°C for around 1 h. Once cold, 1 ml of the neutralizing buffer was pipetted onto the samples in a well plate on ice, and incubated for at least 12 h at 4°C. The neutralizing buffer was then removed, and the samples were washed in PBS 3 times for 15 min and stored in PBS at 4°C thereafter. At this point the samples could be removed from their print substrate as a dense swollen hydrogel, as seen in Figure 2.

### Viscosity

Freshly made solutions of 3 mg/ml collagen type I, 3 mg/ml of collagen type II, and 6 mg/ml collagen type II in 10 mM hydrochloric acid were made by stirring at a low speed at 4°C for at least 48 h. Sonicated samples were treated by pipetting 850 µl of a solution into the vial and sonicating for 5 min, since this the volume normally used for printing. This was repeated 4 times so that there was 3–4 ml of sonicated solution for viscosity measurements. Both the untreated solutions and sonication solutions were tested on an Anton-Paar Lovis 2000 M rolling ball viscosity meter and density meter. In each measurement the solutions, at close to room temperature, were loaded into the 1.8 mm capillary tube already containing a gold ball, using a syringe. The capillary was checked for bubbles then sealed and loaded into the viscosity meter. Of the remaining material in the syringe around 1 ml was inserted into the density meter; a density measurement was required for an accurate viscosity measurement. All measurements were performed at 20°C. A measuring angle of 70° was used for the viscosity measurement.

### Circular Dichroism

CD spectroscopy is particularly effective at detecting changes in collagen structure given the unique spectra resulting from the collagen triple helix with a minimum peak around 198 nm and maximum peak around 221 nm. The presence of randomly coiled denatured collagen should lead attenuation and broadening of the peak around 198 nm, and attenuation or removal of the peak around 221 nm (Greenfield, 2007; Wallace, 2019). Hence, CD was used to characterize any unravelling of the collagen triple helix due to exposure to ultrasonic atomization, or ultrasound alone. For the samples that were exposed to ultrasonic atomization, the set up was similar to a print but with a closed vial to prevent evaporation. Samples that were exposed to ultrasound alone were in a similar set up but with a much smaller starting volume, 150 µl, in the vial which was insufficient to generate the spout needed for atomization. In each case samples were taken at 20, 60, and 120 min. Samples were diluted to 0.15 mg/ml with 0.01 M HCl for the CD measurements. Sample CD spectra were collected between 260 and 185 nm using a Jasco J-1500 CD spectrophotometer with samples in quartz cuvettes with a 1 mm path length. The spectra of the samples were accumulations of 3 measurments between 260 and 185 nm with a scan speed of 20, and 1 nm bandwidth. The spectra of 0.01 M hydrochloric acid was automatically substracted from the spectra by the CD software as background. The ratio of positive to negative (Rpn) peak was calculated to represent any drop in the intensities of characterisitic collagen positive peak at 221 nm, and negative peak at 198 nm, relative to the control. This ratio, Rpn, has often been used as a numerical representation of the changes in collagen CD spectra in response to a denaturing reagent or force (Feng et al., 1996; Feng et al., 1997; Freudenberg et al., 2007).

### Sodium Dodecyl-Sulphate Polyacrylamide Gel Electrophoresis

SDS-PAGE was employed to characterize any degradation of collagen type I or II from ultrasonic aerosolization. Collagen type I and II solutions at 3 mg/ml in 0.01 M hydrochloric acid were treated with 20, 60, or 120 min of ultrasonic aerosolization. These samples and their respective untreated controls of either collagen solution were diluted to 0.3 mg/ml in order to run the SDS-PAGE. The gels were subsequently stained using ProteoSilver stain kit from Sigma Aldrich.

### Nanoindentation

The mechanical properties of the AJP samples were characterized using a Chiaro nanoindenter (Optics11). This ferrule-top nanoindenter and its sister system, the Piuma, have emerged in recent years as useful tools tools for measuring the mechanical properties of hydrogels, and for mechanobiology (Xie et al., 2018; Dobre et al., 2021; Emig et al., 2021). Both systems employ a spherical indenter on a cantilever of known stiffness, which is mounted on a ferrule-topped optical fiber (Chavan et al., 2012). A piezoelectric actuator displaces this assembly, or probe, by a user-defined distance and speed leading to the indenter making contact with the sample and deflection of the cantilever to which it is attached. Interferometry is used to measure the deflection of the cantilever. The deflection is then used to calculate the load, and the stiffness thereafter using a Hertzian (for visco-/elastic material) or Oliver-Pharr (for elastoplastic material) contact model (Mattei and Breel, 2017). While these systems are mostly used to measure local variations in stiffness, they are also used to measure the bulk modulus of hydrogels (Giobbe et al., 2019; Yue et al., 2019; Mattei et al., 2020). This form of micro/nano-indentation has provided results consistent with tensile and compressive test results (Ye et al., 2019). For these measurements a probe with a cantilever stiffness of 0.57 N/m and a 52.5 µm radius spherical indenter was selected. 25 indentations were performed on each sample in a 5 × 5 matrix with 200 µm spacing between indentations in both directions. The indentation profile went to a maximum of 10 µm at an indentation rate of 1.4 µm/s. This indentation included a 4 µm offset from the sample surface to ensure that the probe was not in contact with the material before beginning the indentation.

### Swelling Ratio

Samples were swollen overnight in 1XPBS then blotted on filter paper before weighing for the wet weight. For dry weight measurements, samples were dehydrated in increasing concentrations of ethanol (30, 50, 70, 90% and twice in 100% v/v in ddH_2_O) for 10 min each, and left on aluminium foil to dry overnight.

### Scanning Electron Microscopy

SEM was employed to characterize the layered structure that resulted from the use of AJP. Samples were prepared using a protocol reported by Raub et al. with some modifications (Raub et al., 2007). Briefly, samples were fixed in 4% glutaraldehyde in 1X PBS for 1 h at room temperature, then washed 3 times in PBS for 7 min, and 2 times in ddH_2_O for 7 min. Samples were then dehydrated in increasing concentrations of ethanol in ddH_2_O (30, 50, 70, 90, and 100% v/v) for 10 min each and twice in 100% ethanol. The samples were further dehydrated in increasing concentrations of HMDS in ethanol (33, 50, 66, and 100% v/v) for 15 min each, and twice in 100%. Samples were then torn apart with tweezers and left to dry on aluminium foil under a fume hood overnight. The dried samples were mounted on SEM stubs using carbon tape, and sputter-coated with platinum to a thickness of 5 nm. The samples were imaged at 5 kV.

### Statistical Analysis

Statistical analysis was performed on the nanoindentation and swelling ratio result to discern statistically significant differences between sample groups. In both cases a three-way (collagen type, concentration, and print program) ANOVA was performed with two levels. The values were considered significant if their *p* values were <0.05.

## Results

### Aerosol Jet Printing of Collagen Type I and II

In observing samples printed with program A under the microscope all print lines were seen to overlap, i.e., the print lines of underlying layers could not be observed. Gaps were observed between printed lines in the top layer of samples printed with program B, and print lines of underlying could be observed.

#### Post Print Processing of Aerosol Jet Printed Collagen Type I and II

All samples printed with program A performed as expected for the post-print processing with no signs of dissolution, and clear signs of enhanced structural integrity when handled. However, samples printed with program B appeared thinner, by eye, than those of program A, and some samples appeared to have performed better than others with some samples showing signs of dissolution. However, sample group B still showed signs of structural integrity when handled.

### Viscosity

**FIGURE 3:**
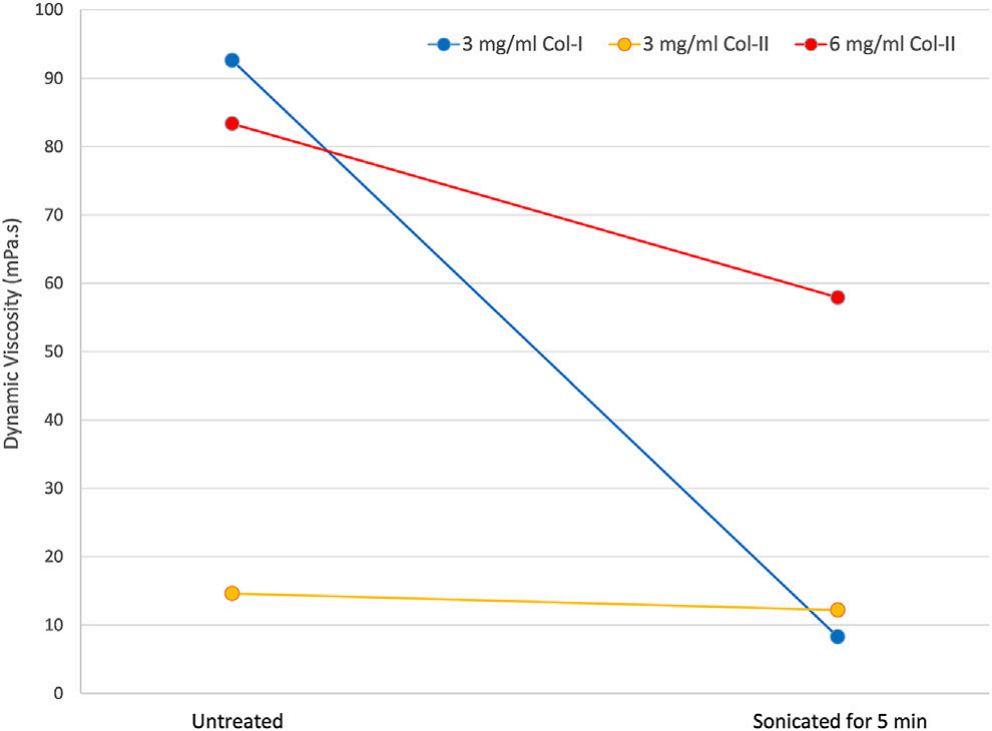
The dynamic viscosity of untreated collagen solutions and the same solutions after a 5-minute treatment with ultrasonic atomization in an aerosol jet printer.

During the treatment with the ultrasonic atomizer all samples generated aerosol soon after initiation of the ultrasound. However, the 3 mg/ml solutions of both collagen type I and type II reached a sufficient rate of aerosol generation for printing almost immediately, whereas the 6 mg/ml collagen type II solution only reached sufficient aerosol generation after around 3 min of the treatment, and the amount of aerosol generated remained low relative to the other solutions. The viscosity of all three solutions dropped as a result of the ultrasonic aerosolization as seen in Figure 3. The most viscous solution, 3 mg/ml collagen type I exhibited the greatest change in viscosity. 6 mg/ml collagen type II also exhibiting a large drop in viscosity whereas 3 mg/ml collagen type II measured a relatively small decrease in viscosity.

### Circular Dichroism

**FIGURE 4:**
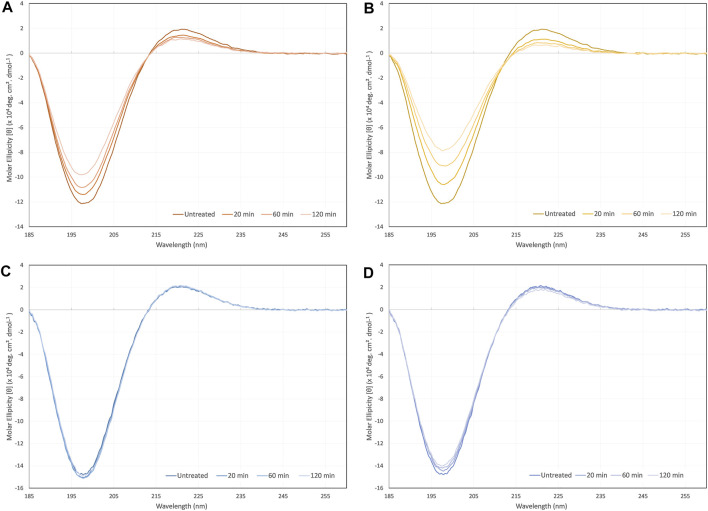
CD spectra of **(A)**collagen type I sonicated non-atomized, **(B)** collagen type I ultrasonically atomized, **(C)** collagen type II sonicated non-atomized, and **(D)** collagen type II ultrasonically atomized.

Typical collagen CD spectra with minima at 198 nm and maxima at 221 nm were measured for all samples, as seen in Figure 4. However, in some cases the sample treatment caused a drop in the intensity of these peaks. Collagen type I was shown to be vulnerable to denaturation as a result of exposure to ultrasonic radiation with a drop in the peak intensities resulting change in Rpn from an untreated control of 0.16, to 0.12 after 2 h of ultrasonic radiation. However, there was no significant red-shift of the crossover point and no clear shift of the 198 and 221 nm peaks. Ultrasonic atomization of collagen type I lead to clearer signs of denaturation with a drop in Rpn from 0.16 to 0.08 after 2 h of atomization, and a gradual red-shift in the cross-over point from 213.6 to 214.9 nm.

Collagen type II showed far less signs of denaturation due to ultrasonic exposure and atomization. The spectra from samples that were exposed to ultrasonic radiation alone were virtually superimposed on each other and resulted in no significant changes in Rpn. Ultrasonic atomization did lead to gradual decreases in the peak intensities at each time point. The Rpn decreased from an untreated control value of 0.14 to 0.13 after 2 h of ultrasonic atomization. There was no significant shift in the crossover point.

### SDS-PAGE

**FIGURE 5:**
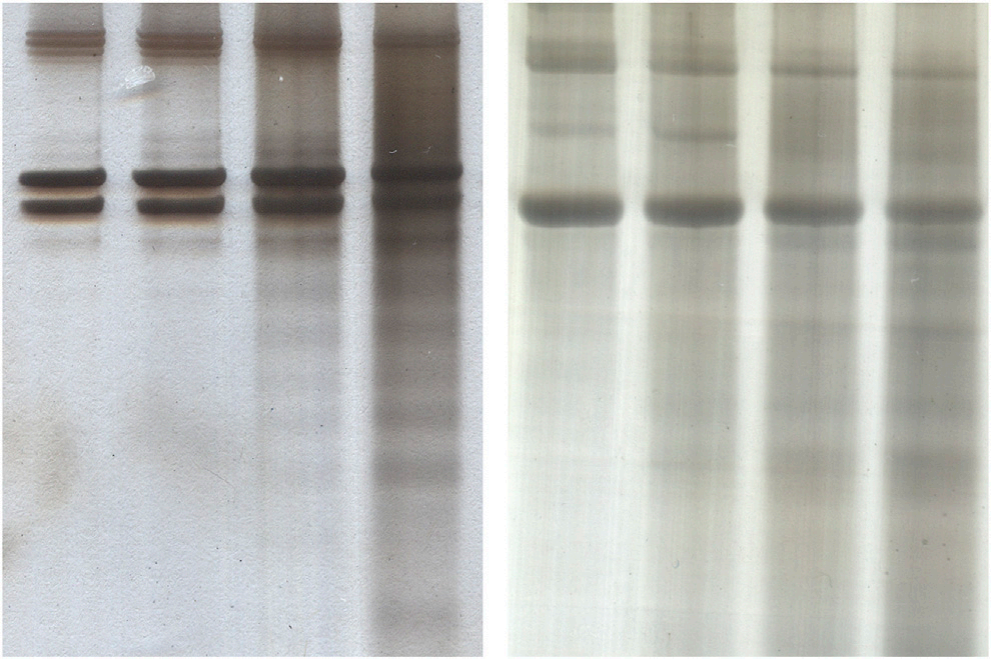
Silver stained SDS-PAGE gels for detecting degradation of collagen of collagen type I **(A)** and collagen type II **(B)** from exposure to ultrasonic atomization. In each case the lanes are in order of (left to right) 0, 20, 60, and 120 min of exposure.

SDS-PAGE revealed that both collagen type I and type II were gradually degraded by ultrasonic atomization as seen in Figure 5. The degraded chains did not appear to show any preferential molecular weight, instead a distribution of what could be confused with non-specific staining spread over the lane. The α and β bands became slightly less intense in both cases after 120 min, and the distribution of degraded α and β chains became increasingly intense in the 60- and 120-min treatment lanes.

### Nanoindentation

**FIGURE 6:**
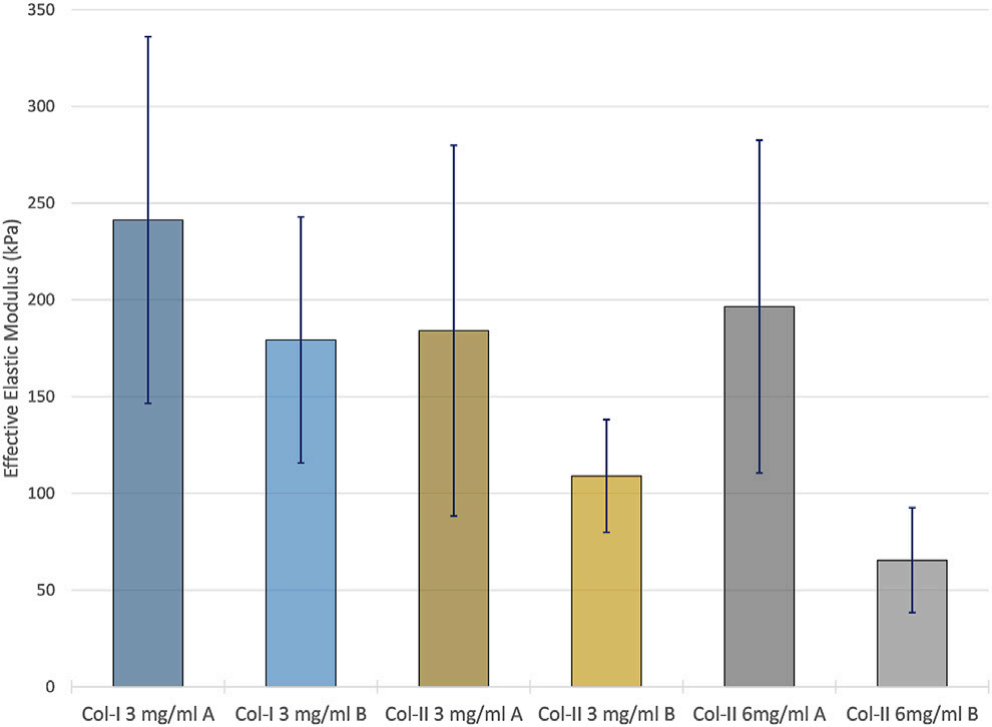
The average effective elastic modulus of each of the sample group measured by nanoindentation using a Hertzian model. Error bars represent the standard deviation of each sample group.

As seen in the nanoindentation results in Figure 6, collagen type I printed with program A resulted in the highest average effective elastic modulus, 241.3 ± 94.3 kPa (±SD), while the 6 mg/ml collagen type II samples printed with program B resulted in the lowest average effective elastic modulus with 65.4 ± 27.1 kPa (±SD). The print program and the collagen type were found to be statistically significant factors in relation to the mechanical properties (*p* < 0.05). Collagen type I samples were stiffer than those made with collagen type II, and samples printed with program A were stiffer than those printed with program B.

### Swelling Ratio

**FIGURE 7:**
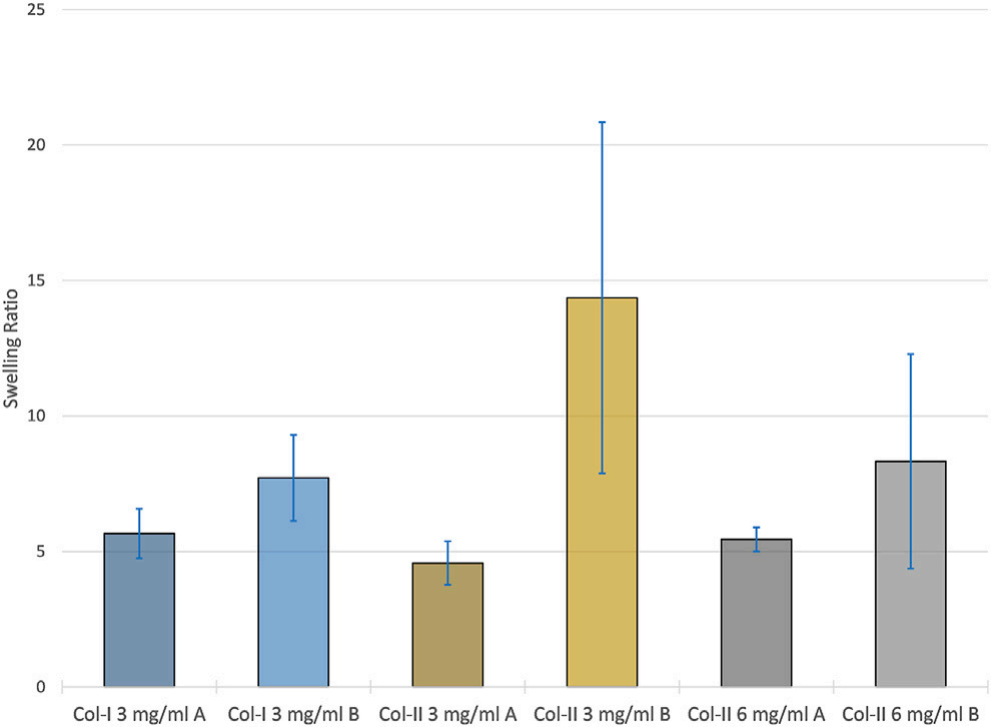
The average swelling ratio of each samples group with error bars representing the standard deviation within each group.

Samples printed with program B had on average had a higher and more variable swelling ratio as seen in Figure 7. The program was found to be the only statistically significant factor in relation to the swelling ratio (*p* < 0.05).

### SEM

**FIGURE 8:**
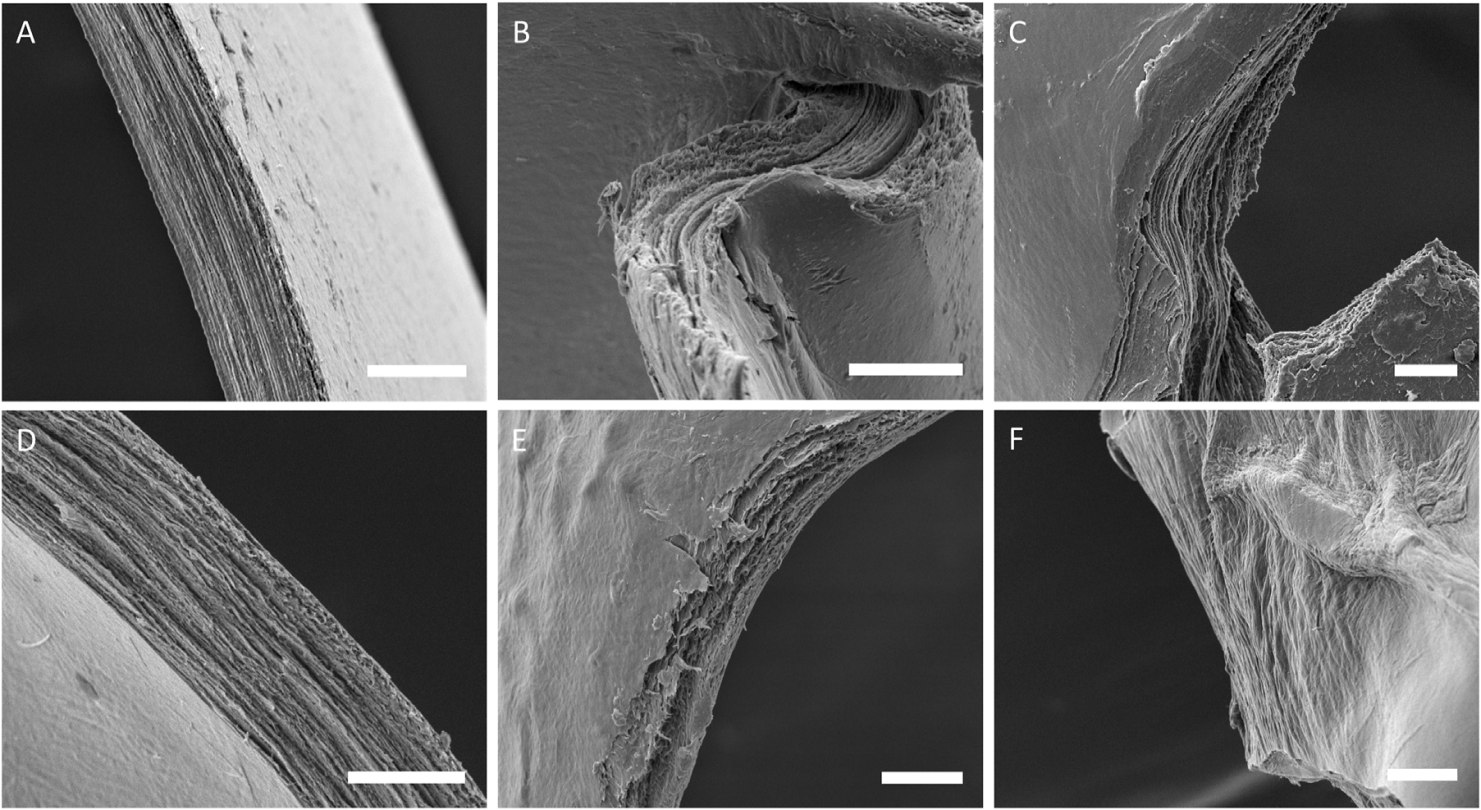
SEM images of cross-sections from sample groups, **(A)** col-I 3 mg/ml A, **(B)** col-II 3 mg/ml A, **(C)** col-II 6 mg/ml A, **(D)** col-I 3 mg/ml B, **(E)** col-II 3 mg/ml B, and **(F)** col-II 6 mg/ml B.

Distinct layers could be resolved in all samples as seen in Figure 8. Whilst in program A samples these layers continued throughout the samples, in samples printed with program B the layers were not always continuous across the section. Most of the samples possessed a very smooth surface topography, however some samples printed with program B had dimples that appeared to continue through several layers.

## Discussion

This is the first research article on aerosol jet printing of collagen type I and/or collagen type II, and can be considered a proof-of-concept study. We have previously reported on aerosol jet printing of recombinant human collagen type III (RHCIII) and highlighted some of the challenges involved, particularly the regarding the neutralisation and crosslinking of AJP collagen (Gibney et al., 2021). Aerosol jet printing of collagen type I and type II has proven to be very similar. Print program A was similar to a program used in to print RHCIII, however here it was printed with higher atomizer flow and higher print velocity in an effort to improve the print time. Print program B was developed to increase the surface area of samples and investigate porous prints, but this was also an effort to improve the print time. By increasing the distance between print lines, the print speed could be increased with a lower risk of what we refer to as “*pooling*”. Pooling is where two or more print lines coalesce leading to the formation of pools which impair print accuracy and can interfere with proceeding layers since they dry slower. Pooling is more likely to occur at corners since the amount of material printed in a given area increases at corners. However, it is likely that some of the significant differences observed between program A and program B samples here, are the result of a less than optimal neutralization process for the program B samples, so this should be kept in mind when considering these differences. It was apparent upon removal of the neutralization buffer that the program B samples had some signs of dissolution. They also appeared far thinner than program A samples which could be due to dissociation of some collagen during incubation in the neutralizing buffer or could be an effect of the printing pattern. It appears likely that the neutralization buffer requires refinement for samples with increased porosity like the program B samples. A higher ethanol concentration in the neutralization buffer could further slow the absorption of the buffer and prevent dissociation of the molecular collagen from the printed material.

The drop in viscosity of the collagen solutions from exposure to ultrasound has been previously reported (albeit for a much lower frequency of ultrasound, 20 kHz) (Giraud-Guille et al., 1994, 2000). The viscosity measurements were performed twice 2 days apart without any change, so the drop does not seem to be transient at least on that time scale. The drop in viscosity is thought to be the result of an initial break up of supramolecular aggregates remaining in solution, followed by a gradual degradation of the collagen as seen in the SDS-PAGE. The shear stress of atomization may also exacerbate this effect of the ultrasound, particularly for collagen type I given the increased unfolding observed for atomized samples versus the non-atomized samples in the CD measurements. The viscosity of the 6 mg/ml collagen type II was considerably higher than the other samples after the atomization treatment, and as noted, there was less atomization occurring for this solution compared to the others. However, the viscosity of the 6 mg/ml would be expected to continue to drop as the atomization time increases. A 6 mg/ml collagen type II solution that was atomized for 1 hour has been measured to have a dynamic viscosity of 7 mPa s. Another explanation for the increased viscosity drop observed for collagen type I solutions compared to collagen type II solutions would be the increased vulnerability of the collagen type I to degradation and unfolding via observed in CD and SDS-PAGE.

It is not clear whether the differences observed between collagen type I and II CD spectra in response to ultrasonic exposure and ultrasonic atomization hold any implications beyond this particular application. The shear forces involved in ultrasonic atomization are likely to be the denaturing factor since ultrasound alone had a less of an impact on the spectra with increasing treatment. There are a number of caveats that must be considered before directly comparing the performance of the two collagen types. The fact that the collagens tested were from different species and from single batches (extractions), thereby makes the findings potentially subject to special variations and batch-to-batch variations. These results were also for monomeric collagen in solution; hence it is difficult to translate any of the findings to assembled collagen fibrils in the ECM. However, even considering these caveats, the fact that ultrasound had no clear impact on collagen type II CD spectra and did on collagen type I spectra is interesting, and at the very least indicates that collagen type II would be more suitable for aerosol jet printing in general. Nevertheless, this does not rule out collagen type I’s suitability for aerosol jet printing, since a large proportion of the collagen must remain in a triple helix conformation for the average signal to still retain the collagen triple helix minimum and maximum peaks.

All aerosol jet printed collagen samples possessed remarkable stiffness for bioprinted collagen constructs that have not been covalently crosslinked. The only significant difference between the sample groups, regarding stiffness, was the print program. However, as stated earlier, the differences observed between the samples printed with the program A or B may have more to do with the neutralization of the construct that any intrinsic properties imparted by the print program. Any significant re-solubilization like that seen in some program B samples would disrupt the layered structure of the printing and thereby change the constructs mechanical behaviour. The average effective elastic moduli of the program A samples were 241.3 ± 94.3 kPa, 185.1 ± 95.8 kPa, and 196.6 ± 86.0 kPa (all ±SD) for 3 mg/ml collagen type I samples, and 3 mg/ml and 6 mg/ml collagen type II samples respectively. The highest reported mechanical characterization results for printed collagen found were compressive moduli of around 110 kPa for glutaraldehyde-crosslinked bovine collagen type I, 47.2 ± 14.2 kPa for EDC-crosslinked porcine-hide collagen, and 21.5 ± 1.4 kPa for porcine-tendon collagen type I without any modification (Lode et al., 2016; Lee et al., 2019b; Osidak et al., 2019). The elastic moduli of the aerosol jet printed collagen constructs printed with program A obtained by nanoindentation were all significantly higher. Furthermore, in separate work where EDC and NHS were added to the neutralizing buffer (40 μg/ml EDC, 24 μg/ml NHS) to crosslink aerosol jet printed 6 mg/ml collagen type II (program A, 576 layers), nanoindentation revealed an average effective elastic modulus of 2.1 ± 0.4 MPa (see supplementary material). These results were compared to results reported on the nanoindentation of dense collagenous tissues. Using a similar Optics11 nanoindenter human ear, septal, and alar cartilage were found to have effective elastic moduli of 1.14 ± 0.71, 2.65 ± 1.78, and 1.26 ± 0.51 MPa respectively (Bos et al., 2018). Similarly, the stroma of human donor corneas were measured to have an average effective elastic modulus of around 75 kPa (Shavkuta et al., 2018). This places aerosol jet printed collagen within reach of replicating the mechanical properties of dense collagenous tissues. Chemical crosslinking could be used to enhance the stiffness of the constructs for closer replication of the stiffest cartilage, whereas different print programs and a deeper understanding of the post-print neutralization could be employed for a closer replication of the weaker collagenous tissues.

For swelling ratio, the only significant difference between sample groups regarding swelling ratio was the print program. Samples printed with program A had a more consistent swelling ratio between and within sample groups whereas program B samples varied a lot between and within sample groups. This could be taken as further evidence of re-solubilization as this would allow collagen to dissociate creating regions within samples with lower density and a higher capacity to absorb water. Evidence of continuous layers traversing entire samples was observed in all program A samples in SEM. However, program B samples possessed regions that appeared amorphous or had very faint signs of layering which were interpreted as signs of re-solubilization. Some program B samples also possessed a strange topography with potholes or dimples which was also interpreted as signs of re-solubilization probably starting at a region where multiple layers were accessible at the surface, like a pore. Nevertheless, the observation of distinct layers in most samples is promising for many applications. We have previously estimated similar layers of RHCIII to be ∼330 nm thick, this would likely increase for the 6 mg/ml collagen type II printing since thickness is usually related to the in concentration in aerosol jet printing. Alternating layers of different material could be used to induce certain behaviours in cells, and layers of conductive material could be printed within a collagen construct for electro-stimulation.

It has been shown in this work that aerosol jet printing can be used to generate stiff collagen constructs with hundreds of layers that are suitable for tissue engineering. While some collagen type I was shown to be denatured by the aerosol jet printing process, the effect was gradual. Hence the denaturation would be mitigated somewhat by the addition of fresh collagen to vial with every hour of printing, as is practice during sample production. Collagen type II was shown to be particularly useful for aerosol jet printing due to its lower viscosity, which allowed a greater concentration range to be printed, and its relative invulnerability to the stresses of the AJP process. While the resolution of this printing process limited scale of the constructs, this was achieved using an aerosol jet printer intended for printing small electrical components with just a few layers. The scalability of this method could potentially be improved by designing an aerosol jet printer with the intention to print biomaterials for biofabrication and perhaps combining this with other bioprinting methods. Moreover, the combination of AJP’s capability to print high resolution structures with conductive inks and it’s demonstrated capability to print collagen will likely be advantageous to the field of electrically stimulated cell culture where inkjet printers have been used to print far more dilute collagen solutions with far fewer layers over conductive material (Weng et al., 2012). The field of bioprinting can only be enhanced with the addition of alternative printing methods and materials, like aerosol jet printing and collagen type II.

## Data Availability

The raw data supporting the conclusions of this article will be made available by the authors, without undue reservation.
