# Supplementary material for: Bioprinting of Collagen Type I and II via Aerosol Jet Printing for the Replication of Dense Collagenous Tissues
Source: Front Bioeng Biotechnol. 2021 Nov 5;9:786945. doi: 10.3389/fbioe.2021.786945 (PMC8602098; doi:10.3389/fbioe.2021.786945)
Supplement: Supplementary file 1 [file DataSheet1.PDF]

Supplementary Material: Bioprinting of collagen type I and II via aerosol jet printing for the replication of dense collagenous tissues

Rory Gibney & Eleonora Ferraris

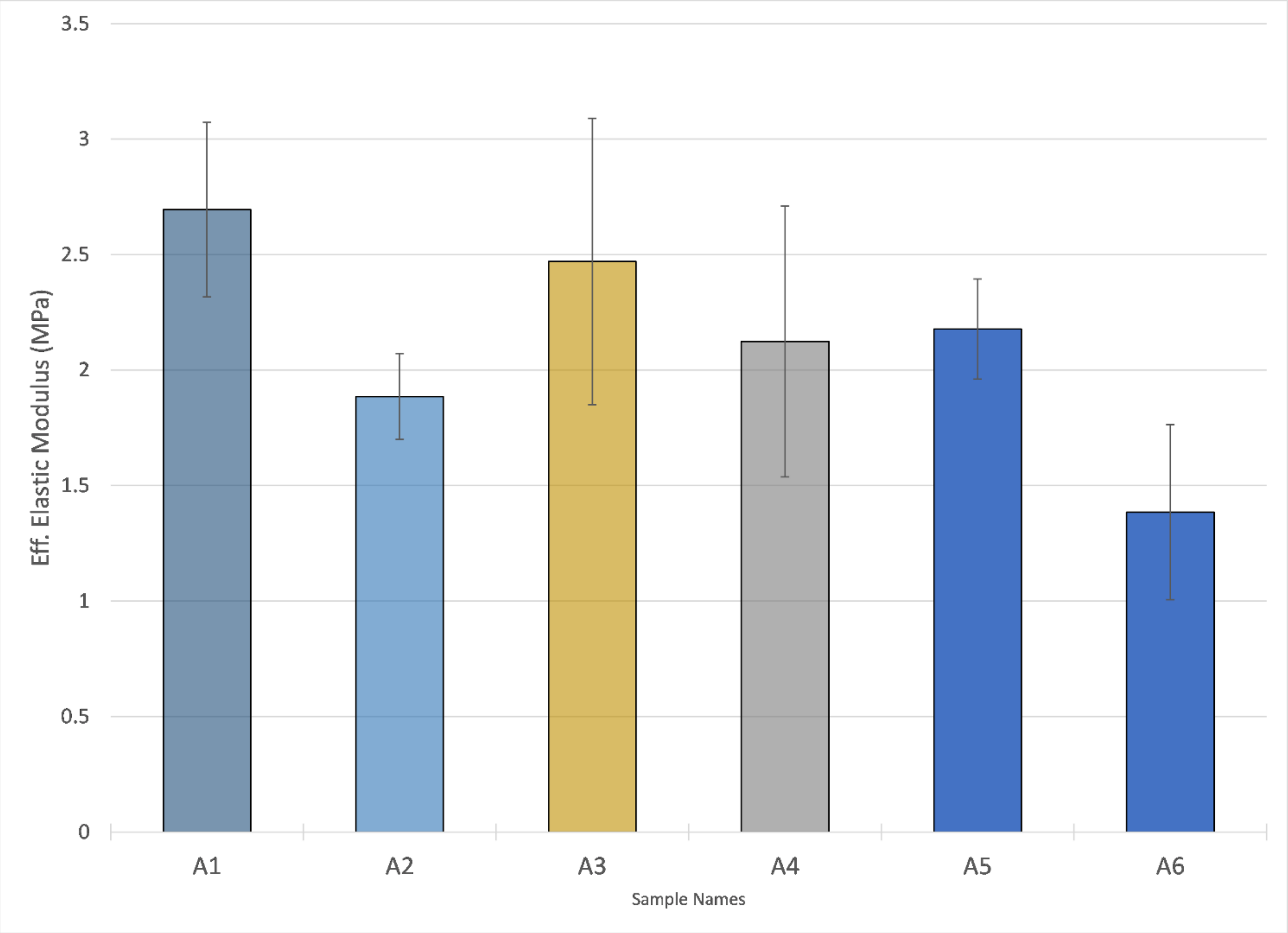

Supplementary Figure 1. Effective elastic moduli measured via nanoindentation of 4.5 mm samples printed using a 6 mg/ml collagen type II solution in 0.01 M HCl and crosslinked in 40 µg/ml EDC, 24 µg/ml NHS. Nanoindentation was performed using a probe with a tip radius of 26.5 µm and a stiffness of 58.7 N/m submerged in 1XPBS. 45 indentations were made in a 3 x 15 matrix with 200 µm spacing in an effort to identify any variation in the sample stiffness between the sample edge and the centre of the sample. Stiffness measurements were consistent throughout samples. The average stiffness of all samples was 2.1 MPa ± 0.4 MPa.
